# Etiological investigation of unintentional solvent exposure among university hospital staffs

**DOI:** 10.4103/0019-5278.75699

**Published:** 2010

**Authors:** Chatchai Ekpanyaskul

**Affiliations:** Department of Preventive and Social Medicine, Faculty of Medicine, Srinakharinwirot University, 114 Sukhumvit 23, Wattana, Bangkok, Thailand

**Keywords:** Building-related illness, hospital, indoor environmental quality, indoor pollution, solvent

## Abstract

**Aim:**

This study was done to investigate unintentional solvent exposure in Srinakharinwirot university hospital staffs with unknown etiology.

**Material and Methods:**

A multidisciplinary investigation was conducted. Total volatile organic compounds (TVOCs) in working environments were measured. Biomarkers of exposure and self-administered questionnaires about clinical symptoms were collected, during and after the incidence, from the affected workers.

**Results:**

The reason behind this event was found to be renovation of the 15^th^ floor. TVOCs contaminated the air hanging unit of the lower 5th floor via space of the pipeline system of the building. The average TVOC value in the complaint area, on the date of notification, was 9.5 ppm. The symptoms and level of hippuric acid, collected during the incidence, were significantly higher than those collected after the problems were solved.

**Conclusions:**

The solvent from the renovation site was a potential source of health hazards for hospital staffs. The relevant authorities should be concerned about implementing a policy for the prevention of indoor pollution in the hospital.

## INTRODUCTION

Nowadays, people are spending more than 90% of their times inside buildings, especially workplaces. The quality of indoor environment is usually neglected, particularly that of hospitals where people are not aware of exposure to chemical hazards.[1–4] Thus, poor indoor environmental quality seems to increase and adversely affect the health and well-being of individuals working in hospitals and other indoor compartments.[5–7] Solvents, particularly toluene, are a common cause for indoor pollution. Several sources can be found such as building activities, furniture and office equipments. It is of particular concern because recent research indicates toluene exposure can result in several toxicities.[8]

Srinakharinwirot University Hospital is located in Ongkharak district, Nakhonnayok province, which is an agriculture area of Thailand. Its 15-floor building is the tallest in the area. The first and second floors are out-patient departments while the third floor is a laboratory unit. The fourth floor has operating rooms. Fifth to seventh floors have secretary offices, library, conference rooms, and classrooms. The in-patient department is on 8–15^th^ floor. The 15^th^ floor, the highest, consists of supporting offices. The building has a central air condition system which is separated into two parts: one is for 1^st^-5^th^ floor while the other is for 6–15^th^ floor.

Early in August 2007, several staffs working on the fifth floor of the hospital notified the occupational health unit. They complained about a foul odor in their working areas every late afternoon for two consecutive weeks with unknown etiology. Some workers also experienced clinical symptoms. This study, therefore, aimed to investigate this event in order to identify the cause and to recommend controls and prevention.

## MATERIALS AND METHODS

A multidisciplinary investigation was conducted by the occupational health team. The concentrations of total volatile organic compounds (TVOCs) in environment were measured by DirectSense TVOC TG502 Multi-Gas photo-ionizing detection monitor. Urine samples for hippuric acid, biomarker of toluene exposure, and self-administered questionnaires for clinical symptoms assessment were also collected at the same time as indoor environmental measurement.

This study was approved by the ethical committee of Srinakharinwirot University. Twenty-seven workers volunteered to participate in this study. Two cases were excluded due to pregnancy and vacation. Of all participants, 72% were females. After signing an informed consent, each worker provided the history of solvent exposure and clinical symptoms. Other confounding factors of solvent exposure and its metabolites were also excluded.[9,10] The urine samples’ and questionnaire collection were repeated 2 months later.

### Statistical analyses

Continuous data were described by the mean and standard error of mean (SEM). A paired t-test was utilized in the comparison between the means in the exposure and nonexposure period. Categorical data were described by frequency and percentage. The Mc Nemar test was utilized in the comparison between proportional data in the exposure and nonexposure period. A *P*-value less than 0.05 was considered statistically significant.

## RESULTS

### Walk-through survey

**Figure 1:**
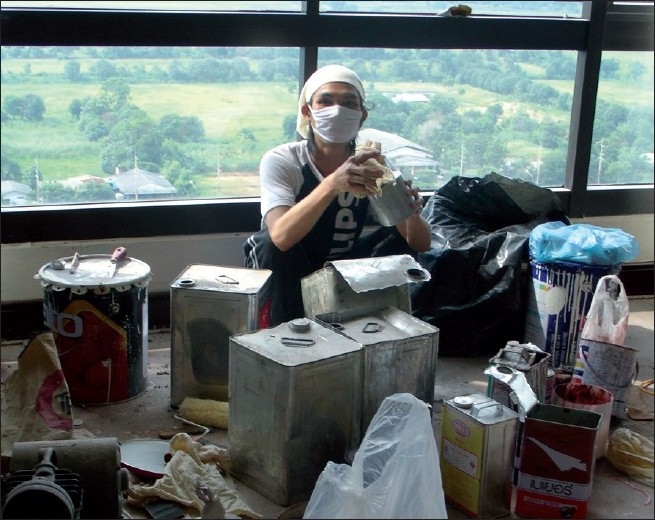
Source of indoor pollution in this event

**Figure 2:**
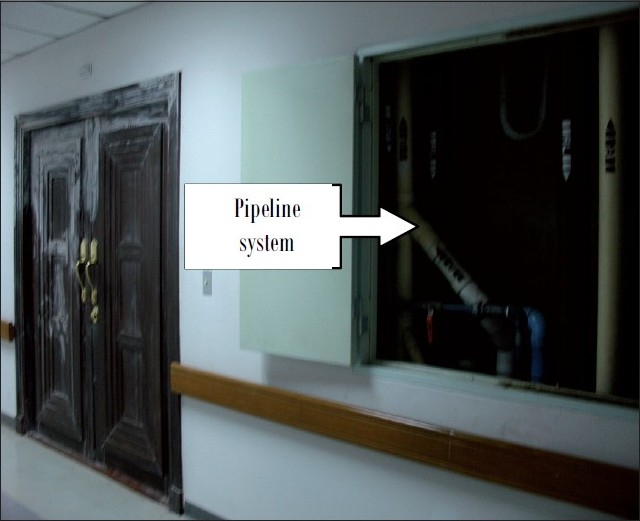
Pathway of connection between 5^th^ and 15^th^ floor of hospital building

The air measurements of all 15 floors showed that only 5^th^ and 15^th^ floors were found to have a thinner odor in working environment. By the process of investigation, we deduced that exposure was caused by the renovation of the royal living room [Figure 1] on the 15^th^ floor. The workers painted and coated doors, wall, and floor with thinner at the same time of detection of odor by affected workers on the 5^th^ floor. The pipeline system connected between each floor [Figure 2] created some unwanted contaminants’ pollution that migrated from the source of the 15^th^ floor to the 5^th^ floor of the building due to the closure of local positive pressure ventilation of the top floor. The polluted air was, thus, returned and moved to the longitudinal space of the pipeline system through even the smallest of opening with differing pressures between the two floors. The solvent was recirculated on the fifth floor and contaminated the air hanging unit (AHU) system which was located between the fifth and sixth floor.

### Indoor environmental quality

The indoor environmental quality was directly measured by DirectSense TVOC devices. The average TVOC level of the area of complaint on the fifth floor on the date of problem notification was 9.5 ppm (range 0–10.5). The TVOC levels measured on the day of data collection and after the incidence were both within normal limit.

### Biomarker of exposure

The urine samples were analyzed for hippuric acid levels by the high-performance liquid chromatography (HPLC) method. We selected this marker because the label of solvent containers which were used for renovation showed the main component as toluene, and urinary hippuric acid at the end of shift during exposure period is helpful for monitoring the acute exposure to toluene. The urinary hippuric acid level during the exposure period ranged from 0.9 g/gCr to 2.0 g/gCr (biological exposure index, BEI, < 1.5 g/gCr).[11] The details are given in Table 1.

**Table 1:** Levels of urinary hippuric acid compared, during and after the incidence

Floor	Zone	Number of subject	Mean ± SEM (g/gCr)		*P*-value
			During the incidence	After the incidence	
5^th^	Library	6	1.60 ± 0.04	0.46 ± 0.06	<0.001
15^th^	Financing, marketing, and medical equipment unit	7	1.40 ± 0.14	0.60 ± 0.10	0.005
	Hospital accreditation and human resource	12	1.56 ± 0.08	0.86 ± 0.12	<0.001
Total		25	1.52 ± 0.06	0.68 ± 0.07	<0.001

### Clinical symptoms

The proportion of clinical symptoms during and after the exposure period, after the exclusion of selected potential confounders, is shown in Table 2. Most of the affected individuals complained of irritation (88%), lethargy, loss of concentration (80%), irritability, and dizziness (72%). However, there was no association between clinical symptoms and the level of urine hippuric acid.

**Table 2:** The number and proportion of subjects who had clinical symptoms, during and after the incidence

Clinical symptom(s)	Number of subjects who have symptoms (%)		*P*-value
	During the incidence	After the incidence	
Loss of memory	11 (44)	5 (20)	NS
Headache	6 (24)	5 (20)	NS
Lethargy	20 (80)	9 (36)	0.003
Loss of muscle power	15 (60)	5 (20)	0.002
Irritation	22 (88)	4 (16)	<0.001
Aggressive mood	15 (60)	3 (12)	<0.001
Loss of concentration	20 (80)	5 (20)	<0.001
Irritability	18 (72)	3 (12)	<0.001
Decreased work performance	12 (48)	7 (28)	NS
Depression	11 (44)	1 (4)	0.002
Anxiety	8 (32)	7 (28)	NS
Visual change	5 (20)	2 (8)	NS
Numbness in palm and sole	6 (24)	3 (12)	NS
Vertigo	7 (28)	3 (12)	NS
Dizziness	18 (72)	5 (20)	0.001
Nausea	13 (52)	1 (4)	<0.001
Paranoid	6 (24)	1 (4)	NS
Coordination imbalance	6 (24)	1 (4)	NS

NS = non statistical significance

## DISCUSSION

This study was an example of an indoor air pollution problem in a hospital. It illustrated the unintentional solvent exposure in indoor environment. Solvents and other chemicals used for renovation and furnishings are a typical cause of indoor pollution. Not only the maintenance or construction workers but also other types of workers could be exposed to solvents. The biological exposure indices of toluene during the exposed period were higher than the levels without occupational exposure and levels in all other more hazardous occupations from previous studies.[8–10,12–16] This level can cause adverse health effects as shown in this study.

For management, during the incidence, our investigation team recommended prevention of pollution by closing the central air condition supply, opening of windows, and increase in local ventilation. We also notified the renovation contractor to change the work schedule from weekdays to weekends. After the investigation, we reported this problem at the hospital’s environment, safety, and occupational health committee meeting. Based on this incidence, the administrative board of the hospital urgently developed an indoor environmental quality policy for the hospital. As a result, a guideline to prevent this similar problem was set up. According to this, renovation, maintenance activities, cleaning, exterminations, and other similar activities known to generate high levels of pollution must be reported to the committee and occupational health unit for risk assessment. All contractors must notify all hospital units of their activities so as to increase awareness. The occupational health unit will also conduct regular monitoring of indoor environmental quality and provide health education to hospital staffs. In addition, the building must be inspected for routine maintenance of air condition and ventilation systems.[17,18]

The major limitation of this study was environmental quality measurement. First, the indoor pollution measurement devices used detected the TVOCs, not the total weight average of the toluene concentration, by a direct reading method. Second, the investigation was done at the final phase of renovation and the exposure of workers might be lower than usual. Third, with a limited budget, no comparison data were collected from the nonexposed workers during the incidence. Last, other toluene metabolites which are more suggestive of low exposure should have been used among the high-risk workers.[14]

Indoor pollution similar to this can occur in other hospitals and workers might develop work-related diseases. All building residents should be concerned and participate in the prevention and control of this problem. They should not generate pollution in indoor environment. Abnormal odor should be reported and investigated. Regular inspection of ventilation systems should be done. The administrative board should implement a policy for indoor environment quality. The occupational health team should integrate this problem with regular health surveillance.[18]

In conclusion, indoor pollution is becoming a chief priority in the control of environmental threats to workers’ health. This investigation suggests that indoor pollution can be a serious problem for indoor environmental quality especially in a hospital. The executive/administrative board of the hospital or relevant authorities should urgently implement a policy for the prevention of indoor environmental quality degradation. The identification and investigation of environmental risk factors, and occupant’s concern, before they become real problems are needed.
